# Patient Selection for Pedal Soft Tissue Augmentation

**DOI:** 10.1093/asjof/ojaa031

**Published:** 2020-06-24

**Authors:** Marissa E Baron, Danielle M Minteer, Beth R Gusenoff, Jeffrey A Gusenoff

**Affiliations:** School of Medicine and the Department of Plastic Surgery, University of Pittsburgh, Pittsburgh, PA

## Abstract

**Background:**

Pedal fat grafting has been shown to improve pain and functional impairment from forefoot fat pad atrophy.

**Objectives:**

The authors aimed to determine if patient demographics and foot characteristics play a role in the level of impact that is achieved following surgery.

**Methods:**

The authors performed a retrospective review of patients who received forefoot autologous fat injections for the treatment of pedal fat pad atrophy. Patient improvement of pain and functional impairment were evaluated for correlation with patient characteristics, including gender, age, BMI, unilateral vs bilateral injections, flexible vs rigid arch, previous foot deformity or surgery, and presence of callus.

**Results:**

Forty-four patients received fat injections into the ball of their foot; 73% of them were women; their mean age was 61 years, and mean BMI was 26.6 kg/m^2^; 75% had injections performed bilaterally; 41% had a flexible arch, 73% had a past history of pedal deformity or surgery, and 43% had callus. Only female gender was found to correlate with an improvement in pain from the time of surgery to 12 months later (*P* = 0.02).

**Conclusions:**

Bilateral rigid, high arched foot type is a risk factor for foot pain and disproportionately represented among these patients. The only patient characteristic found to be correlated with improvement in pain at 12 months post-surgery was female gender. BMI and laterality of injections impacted the course of improvement after surgery. Given current data, all patients with suspected pedal fat pad atrophy should be considered for soft tissue augmentation.

**Level of Evidence: 4:**

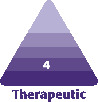

Frequent foot pain is a common problem experienced by approximately 1 in 5 middle-aged and older adults.^1^ It can impact the quality of life and impair daily function. Fat pads in the foot provide protection and shock absorption during the gait cycle. Pedal fat pad atrophy, therefore, causes foot pain and disability as well as an impairment in the ability to perform activities of daily living. Forefoot pedal fat pad atrophy is a diagnosis of exclusion where patients experience foot pain under the metatarsal head without specific parameters for fat pad thickness. Advancing age, obesity, abnormal foot mechanics, corticosteroid injections, previous surgery, and overuse from athletic training or prolonged standing increase risk for developing this condition.^2^

Previous research has demonstrated that autologous pedal fat grafting significantly improves the quality of life for patients experiencing foot pain as a result of pedal fat pad atrophy. Patients express an increase in functional ability, a decrease in pain, and further ability to perform activities of daily living compared with patients receiving standard of care therapy. Furthermore, patients not receiving pedal fat grafting show a worsening of their foot pain and disability over time. Objectively, following forefoot fat injections, fat is retained at long-term follow-up. Increased fat is seen redistributed around the metatarsal head with no increased tissue thickness of the fat pad under the metatarsal head of the forefoot.^3^

Patient selection is an important consideration for all elective procedures. Patients not expected to benefit should, whenever possible, not undergo surgery. In other areas of plastic surgery, such as body contouring, identification of patient characteristics likely to impact success is an integral part of preoperative patient care.^4^ For pedal fat grafting, there has been no previous analysis of which patients see the most improvement and no current report guiding patient selection.

## METHODS

A retrospective review was conducted on previously collected data to determine the impact of patient characteristics and demographics on foot pain and disability following forefoot pedal fat grafting. Typical characteristics seen in patients presenting for foot fat grafting were assessed, including type of arch, differentiation between rigid and collapsible arch, history of previous foot operations (ie, trauma, bunion, hammer toe, neuroma), or presence of callous. Data came from a previous prospective, randomized clinical trial and a case series approved by the University of Pittsburgh Institutional Review Board from January 2014 to September 2017. In both studies, patients provided informed consent and were included if they had foot pain under the head of the metatarsals, were diagnosed with fat pad atrophy by a foot and ankle specialist, and were at least 6 months out from any surgical intervention or injection into the foot. Exclusion criteria included patients with open ulcerations or osteomyelitis, diabetes, active infection anywhere in the body, diagnosis of cancer within the last 12 months and/or presently receiving chemotherapy or radiation treatment, known coagulopathy, systemic disease that would render the fat harvest and injection procedure unsafe to the patient, pregnancy, and tobacco use within the past year. Randomization was conducted by an independent research coordinator not involved in the trial using GraphSoft random number generator software (GraphPad Software, Inc. La Jolla, CA). Patients underwent a surgical procedure as previously described.^2,3,5^

### Individuals

Fifty-one patients with forefoot fad pad atrophy received injections into their forefoot fat pads. Seven patients withdrew from studies before sufficient data being collected for analysis and were excluded. Forty-four patients were included in the final analysis. Demographics and foot characteristics are presented in Table 1. Forty-one patients had data available for comparison at 0 and 6 months, 39 patients had data available for comparison at 6 and 12 months, and 38 patients had data available for comparison at 0 and 12 months. All patients received different amounts of fat based on their individual soft tissue needs with a range of fat injected typically between 3 and 13 mL per side. The technique for fat injection utilized the Coleman method and is described in our previous reports on foot fat grafting.^2,3^ There were no differences between the intervention and control groups in our previous studies.

**Table 1.** Baseline Demographics and Characteristics of Patients Who Received Forefoot Pedal Fat Injections

Variable	No. of patients (%, range)
Number of patients	44
Female patients	32 (73)
Mean age at screening ± SD, yr	61.2 ± 8.7 (43-77)
Mean BMI ± SD, kg/m^2^	26.6 ± 5.5 (19.5-44.6)
Bilateral injections	33 (75)
Flexible arch (vs rigid arch)	18 (41)
History of pedal deformity or surgery	32 (73)
Presence of callus	19 (43)

### Measurement Tool

Foot pain and function were measured using a patient-reported outcome tool entitled the Manchester Foot Pain and Disability Index. This validated assessment tool includes items regarding function, personal appearance, pain, and work/leisure activities.^6^ It was administered on paper at each visit by a member of the research team and answers were subsequently deidentified before analysis. Composite scores assessing pain and function were analyzed with other data not included.

### Statistical Analysis

One-way ANOVA tests were used to examine the relationship between patient characteristics and demographics with change in patient foot pain and function composite survey scores. Patient foot pain and function were examined separately. Changes in composite survey scores were compared across gender, age above or below 65, BMI above or below 25, bilateral or unilateral injections, flexible or rigid foot arch, previous history or no previous history of pedal deformity or surgery, and presence or absence of callus. Changes in survey scores included were from the time of surgery to 6 months post-surgery, time of surgery to 12 months post-surgery, and from 6 months post-surgery to 12 months post-surgery. Change was calculated such that a negative change in survey score indicates an improvement in patient status (ie, a decrease in pain or an increase in function). The Manchester Foot Pain and Disability Index includes 5 items asking about pain and 10 items asking about function. Each item is scored from 0 to 2 based on the answer given. Therefore, the range for possible composite survey scores was from 0 to 10 for pain scores and from 0 to 20 for function scores. All statistical analyses were performed using Stata/SE version 15.1 (StataCorp, Inc., College Station, TX) with statistical significance set to the level of *P* < 0.05.

## RESULTS

**Figure 1.**
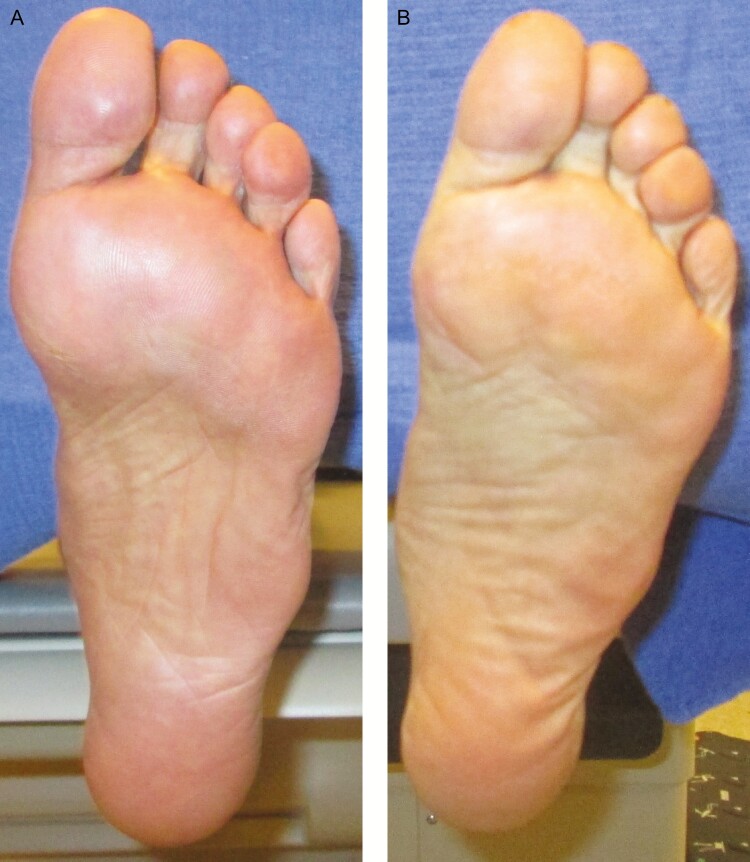
(A) Preoperative photograph of a 63-year-old female (BMI 18.8) with a flexible cavus foot. She has pain on palpation of metatarsal heads 1-5. She had seen 20 foot and ankle specialists over the past 10 years and was diagnosed with fat pad atrophy. (B) Postoperative photograph 12 months after 6 mL of autologous fat was injected into the forefoot.

Patients included in the analysis had a mean age of 61.2 years at the time of screening (range, 43-77 years); 32 patients (73%) identified as female, and 12 patients (27%) identified as male. The mean follow-up time for patients after surgery was 15.4 months (range, 6-24 months). Insufficient data were available to compare outcomes by patient characteristics beyond 12 months. Following the procedure, patients experienced bruising at the donor site, soreness, and pain. No serious adverse events or unanticipated events occurred (Figure 1).

The average total change in function scores from the time of surgery to 12 months was −4.3 with a standard deviation of 5.3 (Table 2). The majority of this change occurred in the first 6 months with an average change of 3.9. The average change in function scores from 6 months to 12 months was 0.4. Female gender, age above 65, bilateral injections, flexible arch, history of pedal deformity or surgery, and presence of callus did not show a significant impact in change in function scores over any time period. BMI over 25 showed a significant difference in change of function scores from 6 months to 12 months with an average difference in scores of −3.1 (*P* = 0.005). There was no significant difference in change in scores from the time of surgery to 6 months or from time of surgery to 12 months among those with BMI above 25 compared to those with BMI below 25.

**Table 2.** Mean Change in Function Scores (± Standard Deviation) by Patient Characteristic From 0-6 months, 6-12 months, and 0-12 months Post Surgery (*P*-Value)

Variable	0-6 months (*n* = 41)	6-12 months (*n* = 39)	0-12 months (*n* = 38)
Total change ± SD	−3.9 ± 4.8	−0.4 ± 3.6	−4.3 ± 5.3
Female	−4.6 ± 5.1	−0.3 ± 2.8	−4.8 ± 5.0
	(*P* = 0.18)	(*P* = 0.75)	(*P* = 0.36)
Male	−2.3 ± 3.4	−0.7 ± 5.5	−3.0 ± 6.0
Age above 65	−4.5 ± 5.2	0.3 ± 1.8	−4.2 ± 5.7
	(*P* = 0.53)	(*P* = 0.33)	(*P* = 0.91)
Age below 65	−3.5 ± 4.5	−0.8 ± 4.3	−4.4 ± 5.1
BMI above 25	−5.1 ± 5.1	1.0 ± 2.7	−4.1 ± 5.9
	(*P* = 0.07)	(*P* = 0.005)**	(*P* = 0.75)
BMI below 25	−2.5 ± 4.0	−2.1 ± 3.8	−4.6 ± 4.5
Bilateral injections	−3.5 ± 4.5	−0.5 ± 3.8	−4.0 ± 5.4
	(*P* = 0.29)	(*P* = 0.70)	(*P* = 0.53)
Unilateral injections	−5.3 ± 5.6	0.0 ± 2.9	−5.3 ± 5.1
Flexible arch	−4.5 ± 4.8	−0.3 ± 3.4	−4.8 ± 5.1
	(*P* = 0.53)	(*P* = 0.92)	(*P* = 0.69)
Rigid arch	−3.5 ± 4.8	−0.4 ± 3.8	−4.0 ± 5.5
History of pedal deformity or surgery	−3.6 ± 4.5	−0.4 ± 3.8	−4.1 ± 5.2
	(*P* = 0.47)	(*P* = 0.88)	(*P* = 0.57)
No history of pedal deformity or surgery	−4.75 ± 5.4	−0.2 ± 2.9	−5.2 ± 5.7
Presence of callus	−4.0 ± 5.2	−0.2 ± 2.8	−4.4 ± 5.0
	(*P* = 0.91)	(*P* = 0.75)	(*P* = 0.94)
No callus	−3.8 ± 4.6	−0.5 ± 4.1	−4.3 ± 5.6

**indicates statistically significant result.

The average total change in pain scores from the time of surgery to 12 months was 2.4 with a standard deviation of 2.9 (Table 3). The majority of this change occurred in the first 6 months with an average change of 2.4. Average change in pain scores from 6 months to 12 months was −0.1. Age above 65, BMI above 25, flexible arch, history of pedal deformity or surgery, and presence of callus did not show a significant impact in change in pain scores at any time point. Female gender showed a significant difference in pain scores from the time of surgery to 12 months with a mean difference in change of scores of 2.5 (*P* = 0.02). Bilateral injections showed a significant difference in pain scores from the time of surgery to 6 months with a mean difference in scores of −2.1 (*P* = 0.03). Bilateral vs unilateral injections did not significantly impact a change in pain scores from 6 months to 12 months or from time of surgery to 12 months.

**Table 3.** Mean Change in Pain Scores (± Standard Deviation) by Patient Characteristic From 0-6 months, 6-12 months, and 0-12 months Post Surgery (*P*-Value)

Variable	0-6 months (*n* = 41)	6-12 months (*n* = 39)	0-12 months (*n* = 38)
Total change ± SD	−2.4 ± 2.9	−0.1 ± 2.3	−2.4 ± 2.9
Female	−2.8 ± 3.1	−0.2 ± 2.2	−3.1 ± 2.8
	(*P* = 0.15)	(*P* = 0.20)	(*P* = 0.02)**
Male	−1.4 ± 2.1	0.9 ± 2.6	−0.6 ± 2.1
Age above 65	−2.9 ± 2.7	0.4 ± 1.3	−2.5 ± 2.8
	(*P* = 0.45)	(*P* = 0.50)	(*P* = 0.90)
Age below 65	−2.2 ± 3.0	−0.1 ± 2.8	−2.4 ± 3.0
BMI above 25	−2.5 ± 3.0	0.4 ± 2.0	−2.2 ± 3.2
	(*P* = 0.84)	(*P* = 0.39)	(*P* = 0.59)
BMI below 25	−2.3 ± 2.8	−0.3 ± 2.7	−2.7 ± 2.5
Bilateral injections	−1.9 ± 2.5	−0.0 ± 2.3	−2.1 ± 2.5
	(*P* = 0.03)**	(*P* = 0.62)	(*P* = 0.28)
Unilateral injections	−4.1 ± 3.4	0.4 ± 2.5	−3.3 ± 3.8
Flexible arch	−3.4 ± 3.1	0.7 ± 2.3	−2.7 ± 3.0
	(*P* = 0.09)	(*P* = 0.18)	(*P* = 0.63)
Rigid arch	−1.8 ± 2.6	−0.3 ± 2.3	−2.2 ± 2.8
History of pedal deformity or surgery	−2.3 ± 2.9	0.4 ± 2.5	−2.1 ± 2.9
	(*P* = 0.72)	(*P* = 0.16)	(*P* = 0.18)
No history of pedal deformity or surgery	−2.7 ± 3.0	−0.9 ± 1.5	−3.6 ± 2.5
Presence of callus	−2.6 ± 3.4	0.1 ± 2.4	−2.8 ± 3.2
	(*P* = 0.67)	(*P* = 0.97)	(*P* = 0.51)
No callus	−2.3 ± 2.5	0.1 ± 2.3	−2.1 ± 2.6

**indicates statistically significant result.

## DISCUSSION

Previous publications have shown the effectiveness of autologous pedal fat grafting at achieving long-lasting improvements in pain and function for patients with pedal fat atrophy.^2,5^ The thickness of the fat pad is initially increased and then returns to baseline with long-term increases in the volume of the fat pad distributed around the metatarsal head.^3^ Other benefits of pedal autologous fat grafting to various areas of the foot have been shown, including improvement of skin quality, treating adult flatfoot deformity, and decreasing the frequency of diabetic foot wounds.^7-9^

There is wide variability in the amount of improvement seen and the course of improvement following surgery. We found a significant difference in the effect of the procedure by patient gender and a difference in the course of improvement by patient BMI. Additionally, the course following surgery was different for patients who received bilateral vs unilateral injections. This suggests that patient characteristics and foot mechanics may be useful in identifying who is most likely to benefit from this procedure and predicting the course and type of improvement they are likely to experience.

Our sample was disproportionately female, appropriately correlating with an increased prevalence of foot pain in women as compared with men.^1^ Additionally, women on average have thinner pedal fat pads than men, a correlation related to their lower height and BMI.^10^ In our analysis, female gender was the only factor found to decrease pain at long-term follow-up. One possible explanation for this effect is an initial thinner fat pad in female patients, providing a higher potential for improvement.

There are 2 hypotheses about the mechanism for the alleviation of pain. The increased volume may offer support despite the absence of increased thickness under the metatarsal head or fat grafting may induce a qualitative rather than a quantitative change in the fat pad, which improves symptoms. Patients insoles are padded after surgery to provide off-loading of the forefoot for 6-8 weeks and they are instructed to avoid strenuous activity. However, we did not totally off-load the grafted region and future studies investigating complete off-loading vs early ambulation are warranted.

The major limitation of this study was the limited sample size. As the evidence grows for the effectivity of autologous pedal fat grafting as a treatment for pedal fat grafting, so does the importance of selecting those patients most likely to benefit and avoiding subjecting to surgery patients unlikely to benefit. However, the limited available data meant that we were unable to fully elucidate which patient characteristics were most significant in effecting the course following surgery. Furthermore, given the retrospective nature of this analysis, we had limited data available on patient demographics and foot characteristics. It is likely that there are influencing factors that were not available to be included in this report. Another major limitation of this study was the subjective nature of the outcome examined. Patient-reported outcomes are an important and popular measure of the study. However, a patient’s perception of their recent functional ability and pain is inherently subjective and vulnerable to bias. We are currently working to establish a reliable foot pain questionnaire specifically for fat pad atrophy. Pedal fat atrophy is a diagnosis of exclusion without specific criteria for tissue thickness; therefore, patients not benefiting may have other biomechanical reasons for their pain.

## CONCLUSIONS

Patient characteristics correlate with the impact of pedal fat grafting surgery and the time course of improvement following surgery. Female gender correlates with improvement in pain at long-term follow-up, while the laterality of injections and BMI impacts the course of improvement following surgery. However, given current data, we advocate for all patients with suspected fat pad atrophy to be considered for soft tissue augmentation. Large-scale studies are called for to further elucidate the impact of various patient characteristics on the probability of success and the course of improvement following pedal fat grafting.
